# Dataset of atmospheric concentrations of polycyclic aromatic hydrocarbons in the Memphis Tri-state Area

**DOI:** 10.1016/j.dib.2023.108923

**Published:** 2023-01-20

**Authors:** Chunrong Jia, Xianqiang Fu, Larry Smith

**Affiliations:** a School of Public Health, University of Memphis, Memphis, TN 38152, USA; b Shelby County Health Department, Memphis, TN 38134, USA

**Keywords:** Polycyclic aromatic hydrocarbon, PAH, PUF sampler, Accelerated solvent extraction, Gas chromatography-mass spectrometry

## Abstract

This dataset contains concentrations (in ng/m^3^) of 32 polycyclic aromatic hydrocarbons (PAHs) in the ambient air in the Memphis Tri-state Area (MTA). In the atmosphere, PAHs are toxic pollutants emitted from incomplete combustion sources. This monitoring campaign was conducted at 19 sites in three neighboring counties in Tennessee, Mississippi, and Arkansas, i.e., MTA, over one year. The monitoring sites represented industrial, urban, suburban, and remote land types. Total suspended particulate (TSP) samples were collected at each site using a high-volume sampler every 12 days from March 13th, 2018, to May 25th, 2019. The collection media consisted of a quartz fiber filter (QFF) and a glass thimble containing polyurethane foam (PUF) and XAD-4 resin that collected particulate- and gas-phase PAHs. Approximately 288 m^3^ of ambient air was drawn over 24 h. The QFF and sorbents were extracted together in an accelerated solvent extraction (ASE) system, and the extract was then nitrogen blown down to 1 ml in an automatic evaporator, and the final extract was analyzed for 32 target PAHs on a gas chromatography/mass spectrometry (GC/MS) system operated in the select-ion-monitoring (SIM) mode. The US Environmental Protection Agency (EPA) reviewed and approved the sampling and analytical protocols. The dataset also has site descriptions, sampling information, and analytical performance. This PAH dataset can be used to explore atmospheric chemistry and sources of PAHs, estimate population exposures to airborne PAHs and the associated health risks, and address environmental health disparities.

## Specifications Table

**Table:** 

Subject	Environmental science
Specific subject area	Air pollution
Type of data	Tables
How the data were acquired	PAHs in the ambient air were collected onto a quartz fiber filter (QFF) and a sorbent “sandwich” containing polyurethane foam (PUF)/XAD-4 resin/PUF, housed in a high-volume sampler (TE-1000, Tisch Environmental Inc., Cleves, OH). The collection media were extracted together in an accelerated solvent extractor (ASE 350, Thermo Scientific, Waltham, MA), and the extract was concentrated using an automatic evaporator (Turbo Vap II, Biotage, Uppsala, Sweden). The final extract was analyzed for 32 target PAHs on a gas chromatography/mass spectrometry (Agilent 7890B/5977A, Agilent Technology Inc., Santa Clara, CA) system operated in the select-ion-monitoring (SIM) mode.
Data format	Raw and analyzed data
Description of data collection	PAH samples were collected at 19 sites in the Memphis Tir-state Area (MTA) every 12 days from March 13^th^, 2018, to May 25^th^, 2019.
Data source location	• Institution: University of Memphis • Region: Memphis Tri-state Area, including Shelby County, Tennessee, DeSoto County, Mississippi, and Crittenden County, Arkansas • Country: USA
Data accessibility	Repository name: Mendeley Data Data identification number: 10.17632/shkhfbh4xd.2 [1] Direct URL to data: https://data.mendeley.com/datasets/shkhfbh4xd
Related research article	C. Jia, Z. Xue, X. Fu, F. Sultana, L.J. Smith, Y. Zhang, Y. Li, B. Liu, Impacts of Independence Day fireworks on pollution levels of atmospheric polycyclic aromatic hydrocarbons (PAHs) in the US, Sci. Total Environ. 743 (2020) 140774. 10.1016/j.scitotenv.2020.140774.

**Value of the Data**

• This is the only community-scale monitoring of ambient PAHs in a metropolitan area in the US in the past decade.
• These data allow quantitative apportionment of spatial and temporal variations in PAH concentrations, which provides bases for designing effective air monitoring programs.
• These data will help address environmental justice issues by examining the relationship between PAH concentrations and sociodemographic factors.
• Researchers can identify major PAH sources, source contributions, and atmospheric PAH chemistry with these data.
• These data can be used in future environmental epidemiologic studies to understand the public health risks associated with PAH exposures.

## 1 Objective

Polycyclic aromatic hydrocarbons (PAHs) are complex chemicals ubiquitously present in the atmosphere. PAHs are known or suspected to cause many adverse health effects, e.g., respiratory diseases, cardiovascular diseases, birth defects, and early childhood development. The US Environmental Protection Agency's (EPA) current monitoring has limited coverage and is insufficient for assessing community-level exposures and health risks. Memphis Tri-state Area (MTA) houses numerous traffic, industrial, fugitive, and natural sources of PAHs and has many health issues that may be related to PAH exposures. MTA covers a gradient of urbanicity, including industrial, urban, suburban, rural, and remote areas. This area also has diverse populations in terms of ethnicity and socioeconomic status. This monitoring campaign in MTA provided valuable data on long-term PAH exposures at the community level. Part of the data has been used to examine the impacts of widespread fireworks on air pollution on Independence Day [2]. For future scientific research, this dataset is expected to have multiple applications in environmental chemistry, exposure and risk assessment, and environmental epidemiology. The speciation of 32 PAHs allows identification and apportionment of sources in this region with techniques such as diagnostic ratios and positive matrix factorization (PMF) [3,4]. The complex, hierarchical study design allows quantification of variance components of the variability, which can guide designing effective sampling plans [5]. The long-term, multi-location measurements represent the best estimation of population exposure to ambient PAHs and allow modeling of individual-level exposures using statistical models such as land-use regressions [6]. The exposure information can then be used for assessing health risks [7] and exploring associations with health outcomes, in particular, children's health [8]. The data will also enhance our understanding of the extent to which minority concentrated communities bear a disproportionate burden of environmental pollution [9,10], in response to EPA's environmental justice movement [11].

## 2 Data Description

All the data for this atmospheric PAHs monitoring campaign are summarized in an Excel file named “Dataset of Atmospheric PAHs in Memphis.xlsx,” freely accessible at https://data.mendeley.com/datasets/shkhfbh4xd [1]. The Excel file consists of six datasheets:

(1) Site_Info: This sheet contains the numbering, identifiers, names, addresses, coordinates, and description of the 19 sites used in this monitoring campaign.
(2) PAHs_Info: This sheet contains the list of 32 target PAHs and their chemical and physical properties.
(3) MS_SIM: This sheet contains the GC/MS SIM settings, including the segments and selected ions.
(4) Cal_1: This sheet contains the performance measures of the first full calibration of the analytical system for target PAHs, including retention times, precision, instrument linearity, and method detection limits (MDLs).
(5) Cal_2: This sheet contains the performance measures of the second full calibration of the analytical system for target PAHs, including retention times, precision, instrument linearity, and method detection limits (MDLs).
(6) Sample_Info & PAH_Con: This sheet contains field and laboratory information on all the samples, including identifiers, sites, start and end times, flow rates, GC/MS analysis filenames, and concentrations (in ng/m^3^) of all PAH samples and quality control samples and analyses.

## 3 Experimental Design, Materials and Methods

### 3.1 Sampling Design

**Fig. 1:**
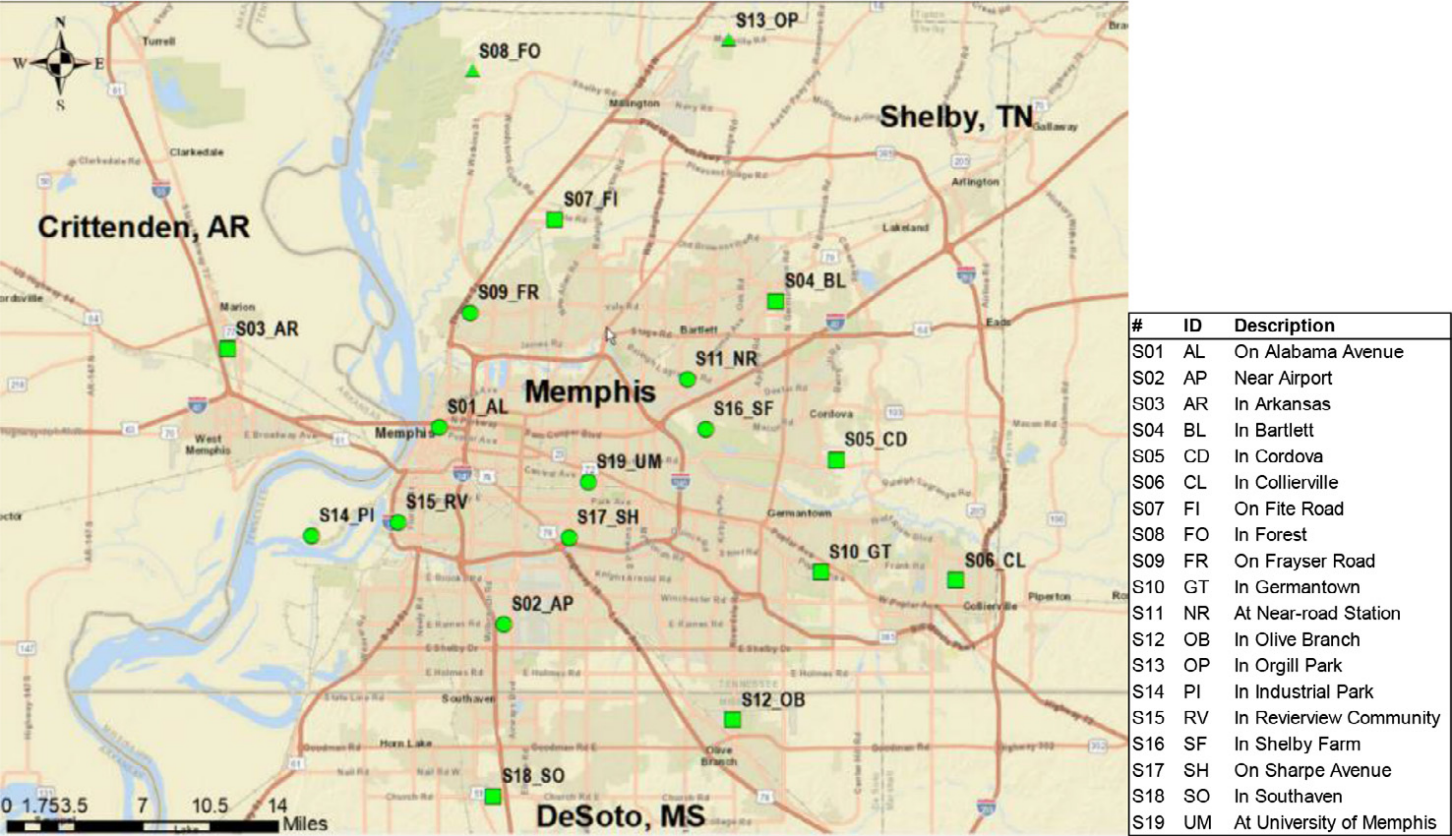
Monitoring sites for the PAH monitoring campaign in the Memphis Tri-state Area.

Air samples were collected in three neighboring counties in MTA: Shelby County, TN, DeSoto County, MS, and Crittenden County, AR (Fig. 1). The land-use type from downtown Memphis displays a clear industrial-urban-suburban-rural-remote gradient, with Memphis as the industrial and urban center. The sampling sites were selected to have broad spatial and temporal representativeness. This campaign recruited 19 monitoring sites, including 16 sites in Shelby County, TN, 2 sites in DeSoto County, MS, and 1 site in Crittenden County, AR (Fig. 1). The site names, surrounding environments, and addresses are listed in the “Site_Info” sheet of the data file. Duplicate samplers were set up at two sites to collect co-located samples and examine field sampling precision. At each site, samples were collected every 12 days to capture all the days of a week. PAH sample collection was performed for 24 ± 1 h.

### 3.2 Sampling Method

PAHs in the ambient air were collected and analyzed following the methods described in EPA Method TO-13A [12] and EPA's Technical Assistance Document for the National Air Toxics Trends Stations (NATTS) Program [13]. No modifications were made to instrumentation or procedures. The same monitoring methods have been widely used by EPA's monitoring network [7] and individual studies in the US [14], [15], [16].

The sampling media consisted of 4-inch diameter, 2 μm pore size quartz fiber filers (QFFs, Part # TE-QMA4, Tisch Environmental Inc., Cleves, OH), pre-cleaned 3/8” diameter polyurethane foams (PUFs, Part #24295, Restek Corporation, Bellefonte, PA), and ultra-clean XAD-4 resin (Part #24230, Restek Corporation, Bellefonte, PA). QFFs were baked at 450 °C in a Muffle furnace (Model: F30420C, Thermo Fisher Scientific, Waltham, MA, USA) for 5 h to remove the potential contaminants. PUFs and XAD-4 resin were cleaned and dried in an accelerated solvent exactor (ASE, Model: ASE350, Dionex / Thermo Scientific, Waltham, MA, USA). All the cleaned sampling media were labeled with the date of cleaning and used within two weeks after the date of cleaning. Before sampling, the glass thimble was prepared by sandwiching approximately 200 g of XAD-4 between two layers of PUF. The front PUF plug was spiked with 50 μL of field surrogate solution that contained 10 ng/μl of fluoranthene-d10 and benzo(a)pyrene-d12.

PAHs in total suspended particulate (TSP) were collected using a high-volume PUF sampler (TE-1000, Tisch Environmental Inc., Cleves, OH). The PUF sampler houses a QFF followed by a glass thimble containing PUF/XAD/PUF sorbents. This sampler was designed to meet the requirements for PAH sampling by EPA Method TO-13A [12]. Before the field sample collection, the PUF sampling flow rates were calibrated following the manufacturer's procedure. The sampler was running at a flow rate of 200 L/min for 24 h, yielding a sample volume of 288 m^3^. Collected samples were stored at -18 °C in a dedicated freezer until extraction and were extracted within 14 days of collection.

### 3.3 Laboratory Analytical Equipment and Reagents

The laboratory analysis of PAH samples followed the principles in EPA's Technical Assistance Document for the NATTS Program [13]. The instruments, chemicals, and reagents for laboratory PAH analysis are summarized in Table 1.

**Table 1:** Equipment and reagents for laboratory PAHs analysis.

Item Description	Vendor	Part#
Equipment		
Accelerated Solvent Extractor	Thermo Scientific	ASE 350
Concentrator	Biotage	Turbo Vap II
GC/MS Analytical System	Agilent Technology	7890B/5977A
Gases		
Helium, UHP	Airgas	HE UHP300
Nitrogen, UHP	Airgas	NI UHP300
Solvents		
Dichloromethane (DCM)	Fisher Scientific	D37-4
Acetone	Fisher Scientific	A18-4
Hexane	Fisher Scientific	H292-4
Standard Solutions		
PAHs Standard Mix	AccuStandard Inc.	H-QME-01
EPA 8270 Semivolatile Internal Standards	SigmaAldrich	CRM48902
PAH Addictions	AccuStandard Inc.	M-8100-R
Surrogate Standard Mix	Restek	31826
Perylene	AccuStandard Inc.	H-121S
Coronene	AccuStandard Inc.	H-116S
Cyclopenta(c,d)pyrene	AccuStandard Inc.	H-242S
Dibenzothiophene	AccuStandard Inc.	H-117S

### 3.4 Sample Preparation

Extraction of samples. For sample analysis, all the collection media were taken out of the freezer and kept at room temperature for 1 h. A sample (or a blank) was spiked with 50 μL of laboratory surrogate solution that contained 10 ng/μL of fluorene-d10 and pyrene-d10. The QFF and PUF/XAD-4/PUF were loaded together in a 100 mL stainless-steel extraction cell, and then extracted in the ASE 350 following an optimized procedure (Table 2).

**Table 2:** ASE 350 parameters for PAH sample extraction.

Parameter	Set point/Value
Solvent Ratio	Hexane: Acetone (v:v)=3:1
Temperature	60 °C
Cycles	3
Purge	60 s
Static time	5 min
Flush	50%

Filtration of extracts. All the extracts were filtered to remove the water content, given the high humidity of air in this region. A filtration funnel was prepared by adding a small plug of deactivated glass wool at the neck and 50–60 g of anhydrous sodium sulfate (10−60 mesh, Fisher Scientific Inc., Waltham, MA) on the top. Each extract was eluted through the filtration materials. After the elution, 30 mL of hexane was eluted to ensure all the analytes were washed out of the filtration materials. The extracts and hexane were collected in a 250 ml pre-cleaned evaporation tube for the final concentration.

Concentration of extracts. The filtered extract was concentrated in an automated solvent evaporation system (TurboVap II, Biotage, Charlotte, NC) with a 1.0 mL endpoint stem. The TurboVap evaporator blew the extract down to 0.3 mL with a gentle nitrogen flow following an optimized procedure (Table 3). After extraction, the extract was transferred to a 2 mL GC autosampler amber vial and added up to the 1 mL marker with hexane.

**Table 3:** TurboVap evaporator parameters for concentrating PAH extracts.

Parameter	Set point/Value
Bath temperature	40 °C
Flow rate	Start at 2.5 mL/min, then up to 3.0 mL/min in 20 min
Total run time	30 min or more, depending on the moisture of the extract

Addition of internal standard (IS) chemicals. IS chemicals were added to all the final extracts before GC/MS analyses to correct for MS variability and potential matrix effects. Each extract was added with 10 µL of IS solution containing naphthalene-d8, acenaphthene-d10, perylene-d12, phenanthrene-d10, and chrysene-d12.

### 3.5 GC/MS Analysis

GC/MS program. The final extract was analyzed on an Agilent 7963A Autosampler-7890B/5977A GC/MS system. The GC housed an HP-5ms Ultra Inert column (30m × 0.25mm ID × 0.25 µm film) with ultra-purity grade (UHP) helium as the carrier gas. A 1.0 μL aliquot of the final extract was injected into GC and analyzed following the program described in Table 4.

**Table 4:** GC/MS operating conditions for PAHs analysis.

Parameters	Conditions
Gas Chromatography	
Column	Agilent Technology, DB-5 ms (0–325 °C; 30 m*250 μm*0.25 μm)
Carrier Gas	Helium
Injection Volume Flow Rate	1 μL, Splitless 1 mL/min, Constant Flow

Temperature Program	
Initial Temperature	70 °C, hold 4 min
Final Temperature	300 °C (20 °C/min to 120 °C, and then 10 °C/min to 300 °C), hold 10 min.
Total Run Time	34.5 min

Mass Spectrometer	
Transfer Line Temperature	290 °C
Source Temperature	230 °C
Electron Energy	70 volts
Ionization Mode	Electron ionization (EI)
Mass Range	40–500 amu, SIM Mode, Time Segments

GC/MS calibration. The initial calibration established a 7-point calibration curve for each target PAH. The standard solutions were prepared in hexane at seven concentrations: 0.02, 0.1, 0.25, 0.5, 1.25, 2.5, and 5.0 μg/mL, equivalent to 0.02, 0.1, 0.25, 0.5, 1.25, 2.5, and 5.0 ng loadings, respectively, with 1 μL GC injection. All the standard solutions also contained surrogate and IS compounds. The solution at each concentration was analyzed twice following the above GC/MS program. Each compound was assigned to the IS compound with the nearest retention time. The MS abundance of a compound was normalized by the abundance of the corresponding IS, yielding an abundance ratio. A linear regression curve was then established by plotting abundance ratios and concentrations. It should be noted that benzo(b)fluoranthene, benzo(j)fluoranthene, and benzo(k)fluoranthese co-eluted at 24.04 min, and thus they were treated as one compound in the calibration.

To determine the method detection limits (MDLs), an MDL sample was prepared by spiking 10 μL of 2 μg/mL PAH mix solution to the sample media, yielding a loading of 20 ng of each PAH. Seven separate MDL samples were then analyzed following the same analytical procedure as a regular sample. The standard deviation (SD) of the seven obtained masses was then calculated. As all the blanks were clean, MDLs were calculated by multiplying SD by the one-sided student's t value at 99% confidence corresponding to the number of spikes analyzed (t = 3.14 for n = 7), i.e.,

(1) $$\text{MDL}=\text{SD}\cdot t_{\left(6,99\%\right)}$$

MDLs were determined annually or when changes to the instrument or preparation procedure resulted in significant changes to the sensitivity of the instrument or procedure.

Calibration verification. A check standard solution containing 0.5 µg/mL of each target PAH were analyzed before the analysis of each batch of samples to verify the initial calibration. The analysis should recover within ± 30% of the nominal concentration.

MS data analysis. The obtained spectra were analyzed for 32 target PAHs in the Enhanced MSD ChemStation (Version F.01.03.2357). A PAH was identified by referring to a combination of the compound's retention time, the m/z ions, and the analyst's experience and judgment. The mass was calculated using the calibration curve.

Calculation of PAH concentrations. The final air concentration of each target PAH was determined by multiplying the concentration in the extract by the final extract volume and dividing by the collected sample volume at standard conditions of 25 °C and 760 mm Hg (STP):

(2) $$C_{A}=\frac{1000\;\times \;\text{Ct}\;\times \;\text{Ve}}{V_{A}}$$

where:

C_A_ = concentration of the target compound in the air (ng/m^3^)

Ct = concentration of the target compound in the extract (μg/mL)

Ve = final volume of extract (mL)

VA = sample volume at STP (m^3^)

### 3.6 GC/MS Sequence

**Fig. 2:**
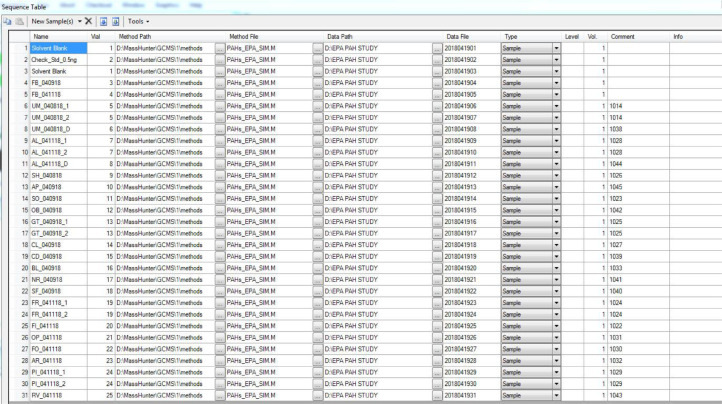
A typical TD-GC/MS sequence established in Markes International Thermal Desorption Control Program (version 5.2.0).

Each round of sampling yielded 21 samples (including two duplicates) and 2 field blanks. A typical GC/MS analysis sequence started with analyses of a solvent blank and a check standard solution. Duplicated injections were made every 5 different samples. A typical sequence consisted of 31 injections/analyses, including 21 samples, 2 field blanks, 2 solvent blanks, 1 check standard, and 5 duplicate GC injections, illustrated in Fig. 2.

## Ethics Statements

This environmental monitoring campaign did not involve human subjects, animal experiments, or data collected from social media platforms.

## CRediT authorship contribution statement

**Chunrong Jia:** Conceptualization, Methodology, Investigation, Resources, Writing – original draft, Funding acquisition. **Xianqiang Fu:** Investigation, Data curation, Formal analysis, Writing – review & editing. **Larry Smith:** Investigation, Project administration, Funding acquisition.

## Declaration of Competing Interest

The authors declare that they have no known competing financial interests or personal relationships that could have appeared to influence the work reported in this paper.

## Data Availability

Dataset of Atmospheric PAHs in Memphis (Original data) (Mendeley Data).
